# Visuo-motor attention during object interaction in children with developmental coordination disorder

**DOI:** 10.1016/j.cortex.2021.02.013

**Published:** 2021-05

**Authors:** Tom Arthur, David J. Harris, Kate Allen, Caitlin E. Naylor, Greg Wood, Sam Vine, Mark R. Wilson, Krasimira Tsaneva-Atanasova, Gavin Buckingham

**Affiliations:** a Department of Sport and Health Sciences, College of Life and Environmental Sciences, University of Exeter, UK; b Department of Health and Social Care, College of Medicine and Health, University of Exeter, UK; c Department of Psychology, University of Bath, UK; d Department of Sport and Exercise Sciences, Research Centre for Musculoskeletal Science and Sports Medicine, Manchester Metropolitan University, UK; e Department of Mathematics, College of Engineering, Mathematics, and Physical Sciences, University of Exeter, UK; f Translational Research Exchange @ Exeter, University of Exeter, UK

**Keywords:** Dyspraxia, DCD, Hand-eye coordination, Perception, Action

## Abstract

Developmental coordination disorder (DCD) describes a condition of poor motor performance in the absence of intellectual impairment. Despite being one of the most prevalent developmental disorders, little is known about how fundamental visuomotor processes might function in this group. One prevalent idea is children with DCD interact with their environment in a less predictive fashion than typically developing children. A metric of prediction which has not been examined in this group is the degree to which the hands and eyes are coordinated when performing manual tasks. To this end, we examined hand and eye movements during an object lifting task in a group of children with DCD (n = 19) and an age-matched group of children without DCD (n = 39). We observed no differences between the groups in terms of how well they coordinated their hands and eyes when lifting objects, nor in terms of the degree by which the eye led the hand. We thus find no evidence to support the proposition that children with DCD coordinate their hands and eyes in a non-predictive fashion. In a follow-up exploratory analysis we did, however, note differences in fundamental patterns of eye movements between the groups, with children in the DCD group showing some evidence of atypical visual sampling strategies and gaze anchoring behaviours during the task.

Developmental Coordination Disorder (DCD) is formally characterised by a broad spectrum of difficulties in performing motor tasks in the absence of any physical, intellectual or sensory impairment. Practically speaking, this means that many otherwise typically developing children struggle with tasks like tying shoelaces, riding bicycles, and catching a ball. DCD is one of the most prevalent, yet understudied, developmental disorders, affecting ~5% of the population (Lingam et al., 2009). Despite intensive efforts from the research community, evidence for a range of interventions is mixed (Eddy et al., 2019; O'Dea et al., 2020; Smits-Engelsman et al., 2018). The difficulty with developing successful interventions for this group likely stems from a lack of understanding about the fundamental mechanisms underpinning DCD.

It has recently been proposed that a selective deficit in the generation or utilization of internal models for feed-forward planning might underpin these motoric impairments (Adams et al., 2014). Evidence for this proposition comes from a number of tasks which have a strong predictive element. For example, van Swieten et al. (2010) conducted a task whereby children with and without DCD had to grasp and rotate an object. In contrast to the typically developing children, children with DCD failed to plan their grasps to maximise their so-called ‘end state comfort’. Furthermore, children with DCD have been shown to be less efficient in their ability to adjust their posture in an anticipatory fashion to compensate for both voluntary and involuntary unloading of force applied to the upper limb (Jover et al., 2010).

Compared to manual actions, eye movements in DCD remain relatively understudied. Recent work has suggested that children with DCD have few fundamental differences in oculomotor control, except in smooth pursuit and anti-saccade tasks (Sumner et al., 2018). In terms of predictive eye movements, children with DCD appear to have impairments in the pre-programmed second saccade of a double-step saccade task (Katschmarsky et al., 2001). Furthermore, it has been reported that children with DCD are relatively poor at synchronizing their eye movements in a predictive fashion to a visual cue they are attempting to track (Langaas et al., 1998) and/or act upon (M. R. Wilson et al., 2013). Recent work examining the causal role of eye movements in the deficits underpinning DCD from Wood et al. (2017) used a randomised-controlled trial approach to demonstrate that the training of saccadic and fixation behaviours during a ball catching task significantly improved the ability of children with DCD to successfully catch a ball. Indeed, this eye movement training has been shown to have a measurable impact on the self-organization of full-body kinematics during ball catching (Słowiński et al., 2019).

Although eye movements themselves are often examined in the context of prediction (Becker & Fuchs, 1985; Diaz, Cooper, & Hayhoe, 2013), hand-eye coordination is fundamental to skilled motor control, with the hand lagging behind the eye in a tightly-coupled fashion during manual tasks (Johansson et al., 2001; Land et al., 1999; Mennie et al., 2007). This lag between the hand and eye is not simply an epiphenomenon of the relative velocities of the hand and eye, but appears to represent a fundamental coupling during manual actions (Fisk & Goodale, 1985). Specifically, predictive eye movements facilitate an earlier ‘anchoring’ of gaze on prospective, goal-relevant action targets (Mennie et al., 2007; Neggers & Bekkering, 2000), such as an object that is about to be lifted (Johansson et al., 2001). This retrieval of advance visual information affords early attention disengagement too, meaning that gaze can shift predictively towards future goal-relevant cues in a sequential fashion (Land et al., 1999; Lavoie et al., 2018). There is emerging evidence that this tight coupling between the hand and eye might be a particularly sensitive index of feedforward sensorimotor control. Indeed, internal action models are proposed to optimise the ‘connectivity’ between hierarchical neurobiological systems for a physiological perspective, see (Friston, 2011), with coherent ‘top-down’ signals modulating both gaze and motor functions (Land, 2009). For example, both the dwell position of the eyes and subsequent kinematics of the hands in an interception task varies as a function of the distribution of the ball's trajectory on previous trials (Diaz, Cooper, Rothkopf, et al., 2013; Mann et al., 2019). These coupled visuomotor signals appear coordinated by common predictive models (Binaee & Diaz, 2019), with hand positions strongly mediated by dynamic gaze behaviours in these tasks.

From a neurodevelopmental perspective, recent studies examining hand-eye coordination in DCD have identified significant impairments in feedforward action control (Warlop et al., 2020; Wilmut et al., 2006; Wilmut et al., 2007; Wilmut & Wann, 2008; P. H. Wilson et al., 2013). When performing visually-guided upper limb movements, such as pointing and reaching actions, children with DCD show delays in attentional disengagement and motor initiation compared to typically developing controls (Wilmut et al., 2006, 2007; Wilmut & Wann, 2008). These atypical visuomotor strategies are not due to any generic deficits in motor kinematics or dynamic attention (Wilmut & Wann, 2008), with hand-eye profiles proving similar between groups during simple object interaction tasks (Sellami, 2008). Instead, the above studies suggest that children with DCD show a selective tendency to utilise feedback-driven control strategies, whereby online visual cues are increasingly sampled at the expense of internal action models (Adams et al., 2014). Although these selective deficits in feedforward control appear to transfer onto complex and/or whole-body visuomotor skills (Wilson et al., 2013b, Wilson et al., 2013a; Warlop et al., 2020; Parr et al., 2020), much is left unknown about how hand-eye coordination unfolds in naturalistic tasks in this population.

In this study, we present data on the eye movements and hand kinematics of a sample of children with and without DCD in a simple object lifting task. This study was conducted primarily in the context of understanding how prior expectations might impact perception of object weight and the fingertip forces used to interact with objects (Allen et al., in preparation). Despite these challenging conditions for measuring gaze behaviour, we were able to successfully measure hand-eye coordination in a subset of our participants during the lifting phase of the task, allowing us to shed further light on how indices of this predictive hand-eye coordination might relate to the sensorimotor differences in DCD. In addition to being a commonplace daily activity undertaken from early childhood, interacting with an object is a particularly interesting behaviour as it is not challenging or frustrating, making it well-suited for examining sensorimotor prediction in neurotypical and clinical populations (Arthur et al., 2019, 2020).

Our working hypothesis is that, if DCD is driven by atypicalities in predictive behaviours related to hand-eye coordination, children with DCD will show lower levels of coupling between the eye and the hand than children without DCD when lifting objects in a visually-guided fashion. Here, a tendency to utilise feedback-driven sensory cues would be expected to disrupt the close spatiotemporal relationships that are typically afforded between gaze and motor signals during ‘top-down’ action control (see Land, 2009). On this basis, we also expected children with DCD to show a shorter lag between the eye and the hand, indicative of a less predictive and more feedback-driven visuomotor strategy.

## 1 Materials and method

### 1.1 Participants

121 children aged between 8 and 12 years were recruited from the south west of England through school visits, word of mouth, and social media advertisement. We aimed to recruit 60 children with DCD, and 60 children without DCD as part of a large project examining multiple facets of sensorimotor prediction in this population. The initial criteria for inclusion in the DCD group were made by parental assessment that their child has movement difficulties. Parents then were asked to complete the revised version of the Developmental Coordination Disorder Questionnaire (DCDQ) – a well-validated 15-item questionnaire for parents to fill out which will provide an initial quantitative assessment of whether their child is likely to have DCD (Wilson et al., 2009). Parents also confirmed that their child did not suffer from any general medical condition known to affect sensorimotor function (e.g., cerebral palsy, hemiplegia, or muscular dystrophy) and had no diagnosis of learning difficulties. If the child fell within the recommended scoring range of 15–55, they were invited to come to the laboratory to take part. Once in the laboratory, children were administered the Movement ABC-2 assessment battery (Henderson et al., 2007), which was used to assign participants to the DCD or the Control groups, independent of the parental assessment. While data collection was ongoing, parents also completed computer versions of the Autism Spectrum Quotient: Children's Version (Auyeung et al., 2008) to assess autistic-like traits, in addition to the ADHD rating Scale-IV (Pappas, 2006) to assess traits associated with attention deficit and hyperactivity disorder.

Extensive data cleaning and exclusion due to data loss (outlined in detail below) yielded a final sample of 70. Following the protocol of Wood et al. (2017) the DCD group were defined as those individuals who scored at, or lower than, the 5th percentile (n = 19). The Control group were defined as those who scored above the 15th percentile (n = 39). The 12 participants whose MABC-2 scores fell between the 5th and 15th percentile were removed from the main analysis comparing groups, but included in the follow-up correlational analysis. Demographic data for each group is outlined in Table 1. No part of the study procedures or analyses was pre-registered in a time-stamped, institutional registry prior to the research being conducted. We report how we determined our sample size, all data exclusions (if any), all inclusion/exclusion criteria, whether inclusion/exclusion criteria were established prior to data analysis, all manipulations, and all measures in the study.

**Table 1:** The average (SD) demographic scores of the DCD and Control groups.

Group	Mean age (years)	Gender (count Male/female)	Preferred hand (count right/left)	MABC-2 (standardized score)	DCD-Q score	AQ score	ADHD score
DCD (n = 19)	9.7 (1.2)	15/4	17/2	3.4 (1.5)	33.3 (13.9)	70.3 (27.2)	24.5 (12.1)
Control (n = 39)	9.6 (1.1)	19/20	35/4	10.3 (2.4)	58.8 (12.5)	52.8 (16.4)	13.7 (9.0)

### 1.2 Stimuli and equipment

Participants lifted five black plastic test cylinders, fabricated with a mount on the centre of the top surface to allow for the rapid removal and replacement of a lifting handle. The cylinders were all 7.5 cm tall, with a ‘medium-sized’ cylinder with a diameter of 7.5 cm and a mass of 490 g, a pair of ‘small’ cylinders with a diameter of 5 cm and masses of 355 g and 490 g, and a pair of ‘large’ cylinders with a diameter of 10 cm and masses of 355 g and 490 g.

A Pupil Labs mobile eye-tracking system (Pupil Labs, Sanderstrasse, Berlin, Germany) recorded participants’ eye movements using scene and infrared eye camera footage. This eye-tracking system comprised a pair of lightweight glasses (34 g) which calculated gaze positions at 90 Hz with a spatial accuracy of ± .60° and precision of .08° (Kassner et al., 2014). The eye-tracking system was calibrated before lifting trials, and upon any displacement of gaze cameras during the testing session using the native Pupil Labs screen marker routine on an LED monitor (60.96 cm; Dell Computer Corporation, Round Rock, TX, USA). The monitor was placed directly in front of participants, so that it spanned the two-dimensional (picture plane) task workspace from their perspective. This meant that gaze could be specifically calibrated in relation to the current and future position of the lifting object (see Arthur et al., 2019 for more details).

Upper-limb kinematics were measured at 120Hz with an 8-camera Optitrak Flex 13 motion capture system. This system recorded the positions of 5-marker rigid bodies attached to (1) the top surface of the object being lifted, (2) the wrist of participants’ preferred hand, and (3) the eye tracker in three dimensions. Wrist and object kinematics were extracted, and the x, y, and z position vectors were combined to yield resultant position for each rigid body.

Finally, three-dimensional fingertip forces were measured at 500hz with an ATI Nano17 forces sensor mounted into a custom-made aluminium and textured plastic lifting handle. Grip force was defined as the force applied orthogonal to the handle. This metric was not examined in the current manuscript (see Allen et al., in preparation), but was used for the data pre-processing and synchronization as outlined below.

### 1.3 Procedure

Upon arriving at the laboratory, children and parents were provided with a participant information sheet (which they had also received at least 48 h prior to their visit) and were given the opportunity to ask any additional questions. Parents then provided informed written consent and children provided written assent to take part in the study.

Following the consent procedure, the child, accompanied by a parent, moved to a separate room and completed the Movement ABC-2 assessment battery (MABC-2; Henderson et al., 2007). The MABC-2 was used to assess movement capabilities and assign participants to the DCD or the Control groups, independent of the parental assessment (Table 1). Legal copyright restrictions prevent public archiving of this test battery, which can be obtained from the copyright holders in the cited references.

Children and parents were then invited into a laboratory to take part in the main object lifting experiment trial. Children were asked to sit opposite the researcher at a large desk, while parents were invited to sit in the same room to offer support, but to remain out of the child's sight where possible. The researcher introduced children to the equipment they would be using during the experiment including the motion tracking cameras and the eye tracking glasses. Before setting up this equipment, the researcher explained the lifting task to the child using a standardised script:

*“For the next bit of the study I'm going to ask you to reach out and pick up a number of objects over and over again. To do this I'd like you to sit with your hands resting on the table and focus on the sticker in the middle of the clapboard. I'll press some buttons on the computer and then you will hear a beep. When you hear the beep I'm going to open the clapboard and I want you to reach out and pick up the object using your thumb and first finger in a smooth, controlled and confident fashion. Lift the object a short distance off the table and hold it steady until you hear a second beep. When you hear this second beep put the object back down and pop your hands back on the table.*

*After you've lifted the object, I'm going to ask you to give me a number to tell me how heavy you thought it felt. You can use any scale you like so 1 to 10 or 1 to 100 as long as big numbers mean heavier feeling objects”*

Children then completed five practice trials. The practice trials involved lifting the same medium sized cylinder for each trial and allowed the child the opportunity to become familiar with the object lifting procedure.

After the practice trials, the researcher set up the motion tracking and eye tracking ready for the main experimental trials. For the motion tracking, this involved placing a motion tracking wrist band on the child's dominant hand and checking this was being picked up by the Optitrack cameras. For the eye tracking, this involved placing the eye tracking glasses on the child and adjusting the cameras to ensure they were capturing the eyes sufficiently. Where children were wearing glasses, they were asked to remove these provided they felt comfortable and able to do so without considerably affecting their vision. The researcher then conducted the calibration process which involved asking the child to remain still and to focus their gaze on 10 dots which appeared consecutively on a computer screen (see below). The researcher assessed the reliability of the calibration by asking the child to fixate different corners of the monitor. Where the calibration was unsuccessful, the child was asked to repeat this process until the eye tracking was deemed acceptable.

**Fig. 1:**
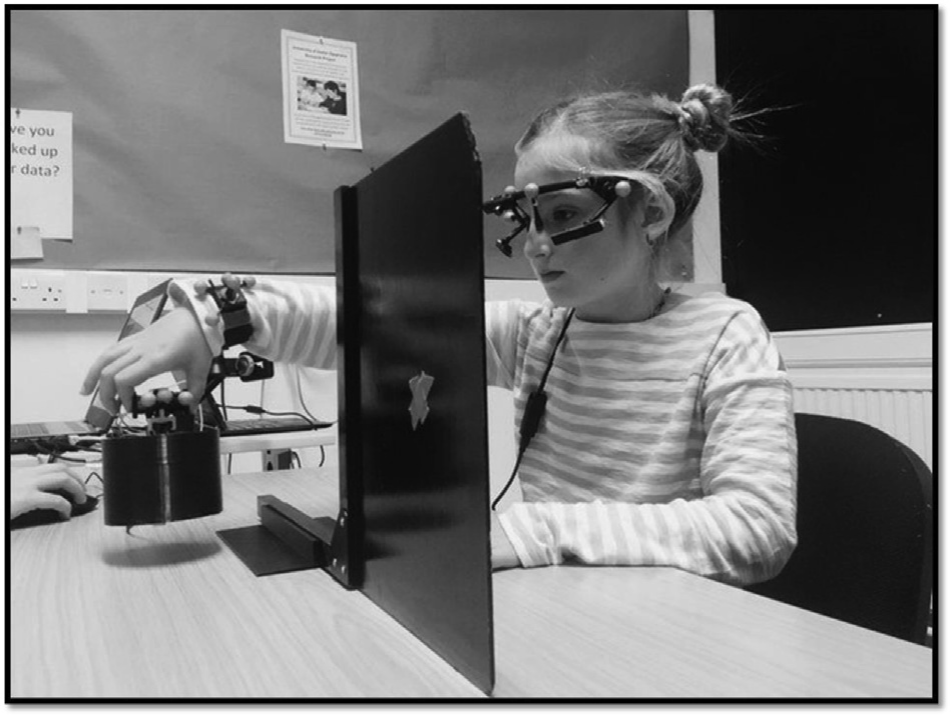
Demonstration of the task, with the participant lifting the object with their preferred hand when the clapper board was opened.

The experimental trials then began (see Fig. 1), with five consecutive lifts of the medium-sized cylinder (as was used in the practice trials) followed by 8 lifts apiece of each of the four large and small cylinders. These larger and smaller cylinders were lifted in one of three randomly-generated orders for a total of 37 lifts. The participant's hand and eye position, in addition to their fingertip forces, was recorded on each lift, along with the verbal heaviness rating (to be presented in Allen et al., in prep).

During the object lifting data collection, parents completed computer versions of the Autism Spectrum Quotient: Children's Version (Auyeung et al., 2008) to assess autistic-like traits, in addition to the ADHD rating Scale-IV (Pappas, 2006) to assess traits associated with attention deficit and hyperactivity disorder on a computer in the corner of the laboratory.

### 1.4 Data analysis

#### 1.4.1 Kinematic data

Positional data for the rigid bodies attached to the wrist and object were smoothed using a dual-pass, zero-phase lag Butterworth filter at 10 Hz (Franks et al., 1990), before being resampled at 90 Hz (i.e., the frequency of gaze recording). The velocity of these signals was then calculated from the differences in average rigid body position between samples. From here, movements were identified using a 50 mm/sec velocity threshold (as in Arthur et al., 2019). Specifically, the reach movement started when hand velocity first exceeded 50 mm/sec for three consecutive frames (Eastough & Edwards, 2007) and ended when the fingers contacted with the force sensors (grasp onset). The lift movement started at grasp onset and finished at the frame where the object velocity dropped below 50 mm/sec for three consecutive frames (lift offset; Arthur et al., 2019). For exploratory analysis of the reach kinematics (see Supplementary Materials), the total movement duration (s), maximum velocity of the hand, and time to peak velocity (% of reach movement time) were averaged for each participant.

#### 1.4.2 Gaze data

Eye-tracking data were smoothed using a dual-pass, zero-phase Butterworth filter at 30-Hz. This low-pass cut-off threshold has been widely used for detecting gaze velocity metrics in previous sensorimotor studies (e.g., Fooken & Spering, 2019), and was thus deemed appropriate for the analysis of the predictive, goal-driven eye movements in this task. The velocity and acceleration of these filtered signals were then calculated from the distance between samples. For all trials, we visually inspected the eye-tracking video footage, using Pupil Player software (Pupil Labs 2016), to manually identify trial onset and offset. For the main analysis, which focused on the lift phase, data were segmented from the first moment of contact between the hand and object handle (i.e., grasp onset), to the frame where participant finished lifting the object to its peak (lift offset). Conversely, for the supplementary reach phase analysis, we examined data that preceded grasp onset, with trial onset representing the first frame in which the lifting object became visible to the participant.

Following this manual processing, the frequency of gaze saccades and fixations were inspected during these time periods. Saccades were identified from portions of data where gaze acceleration was more than five times its median absolute acceleration (Mann et al., 2019). To avoid erroneous detections (e.g., due to blinks, tracker-noise artefacts), gaze acceleration had to exceed this threshold for five consecutive frames and was not preceded or followed by missing data. Conversely, a spatial dispersion algorithm was used to calculate gaze fixations (Krassanakis et al., 2014). Here, fixations were defined using a 1° spatial dispersion threshold, with a minimum duration of 100 msec applied (Salvucci & Goldberg, 2000). The frequency of saccades and fixations per second during the distinct reach phase and during the lift phase of the trial were averaged for each participant, to provide an overview of visual sampling behaviour. Moreover, to inspect gaze anchoring during the reach phase (see Supplementary materials), we also inspected the timing of participants’ final visual fixation that was made before making contact with the object. The onset of this gaze event, relative to reach onset time, was then averaged for each participant, using the median across all trials.

#### 1.4.3 Hand-eye integration

When lifting objects, vertical hand and eye movements typically display similar positional profiles over time (Johansson et al., 2001), meaning that the integration and time ‘lag’ between these signals can be examined using cross-correlational analysis (Arthur et al., 2019; Chattington et al., 2007). As such, hand and eye movement signals were synchronised for time, by matching the grasp onset frame within each data series. To do this for the hand data, we identified the frame denoting the onset of grip force (>1 N) using an ATI Nano-17 force transducer inbuilt within the object lifting handle. As these force data were time-synchronised to the raw kinematic data, this grasp onset frame could be annotated and matched with the corresponding timepoint in the visually-inspected gaze video footage. Both movement signals were subsequently segmented from grasp onset until the lift offset frame (see above), and cross-correlations were examined using a custom algorithm in MATLAB (available at https://osf.io/fm247/). As such, only the grasp and lift phases were analysed, since vertical hand and eye movements follow similar positional changes over time during these portions of the trial (see Arthur et al., 2019 supplementary analysis).^2^ The resulting cross-correlogram identified the peak covariation between vertical hand and eye signals (i.e., peak R) and the ‘lag’ (converted into time) for when this peak occurred. The peak R value illustrated how well these signals matched during each trial (once offset in time), with more integrated hand-eye patterns corresponding to higher index values (i.e., closer to one). Conversely, the time ‘lag’ value indicated the degree to which one signal led another (in seconds), with positive scores signifying that eye movements were preceding the hand (and vice versa). These time-domain variables were then averaged for each participant, using the median across all trials (as in Mann et al., 2019).

#### 1.4.4 Data treatment and analysis

Due to an inability to wear the eye-tracker for prolonged periods, difficulties with calibration, or a clear lack of attention on task across multiple trials, we conducted extensive data cleaning and verification protocols. This involved one of the authors watching a video of each trial from the participant's perspective from the head-mounted camera attached to the eye tracker. The researcher used a qualitative coding system of 0–4 to judge the quality of data in each trial. Invalid trials whereby the lifting procedure was not correctly followed were given a 0. Trials in which the quality of eye-tracking was too poor to see any useful patterns were given a 1. Useful trials in which patterns of eye-movement and lift were clear were given a 2 if there were quality issues (such as tracker flickering or disappearance) but given a 3 if the issues were very minor. All trials which had no clear tracking or procedural issues were given a 4. Example videos for each code were saved as a reference to remain consistent throughout the coding process, and thorough notes were kept to ensure the coding system was well-defined. Furthermore, any unusual observations in specific trials (such as offset gaze positions which appeared to be due to tracking error) were recorded to help with further exclusions.

All trials that had a data quality score of 0, 1 or 2 were removed from the final sample. After these exclusions, any participants who had fewer than 18 valid trials remaining (i.e., 50% of a complete dataset) were excluded from further analysis. These procedures yielded a sample of 73 individuals. From these, 3 datasets were also removed due to errors in kinematic data collection, yielding a final sample of 70. A file detailing the scoring and exclusions for each participant can be found at: https://osf.io/fm247/.

Data analysis was conducted in JASP (v0.12.1) (Love et al., 2019). Data were first screened for outlying values more than 3 standard deviations from the mean. Outliers were replaced with a Winsorized score by changing the value to a value 1% larger (or smaller) than the next most extreme score. Bayes factors were calculated for all *t*-tests using a default Cauchy prior. We report BF_10_ and take BF_10_ < 1/3 as evidence in favour of the null, and BF_10_ > 3 as evidence for the alternative (van Doorn et al., 2019). All data are available at: https://osf.io/fm247/.

## 2 Results

### 2.1 Sample

We first investigated the degree to which our DCD and Control groups differed in their motor behaviour by comparing the scores on the MABC-2 test undertaken by participants in the lab, and the retrospective reports of participants’ parents on the DCD-Q. This analysis, of course, has no value for hypothesis testing due to the sample being divided on one of these metrics, but does serve to provide an indication of the magnitude of difference which might be expected across the novel measures outlined below. As can be seen in Table 1, children with DCD were scored significantly lower than children in the Control group on both the MABC-2 [t(56) = 11.4, *p* < .001, d = 3.2, BF_10_ = 1.25e+13] and the DCD-Q [t(56) = 7.0, *p* < .001, d = 1.7, BF_10_ = 2.49e+6].

### 2.2 Qualitative description of data

Prior to quantitatively examining the metrics of hand-eye integration and visual sampling behaviour which could be extracted from our eye-tracking and motion capture variables (see sections below), it is first worth describing participants' general gaze responses in this task, which showed a number of qualitatively consistent features. As described in our previous adult studies (Arthur et al., 2020), most participants tended to ‘anchor’ their gaze upon the stationary lifting object during the reach and grasp phases of the task. Thereafter, during the lifting action itself, participants tended to use a combination of pursuit and saccadic eye movements to track the object's in-flight trajectory. Finally, upon reaching a stable ‘hold’ position, object-directed fixations were then resumed, often intermittently, until the offset of the trial. Such gaze patterns are consistent with previous studies (Johansson et al., 2001; Arthur et al., 2019, 2020), and are said to be ‘supervised’ by feedforward action schemas (Land, 2009). Therefore, any DCD-related impairment in this use of these internal predictive models could be expected to alter the integration of visual and motor signals (see below).

### 2.3 Main analysis

**Fig. 2:**
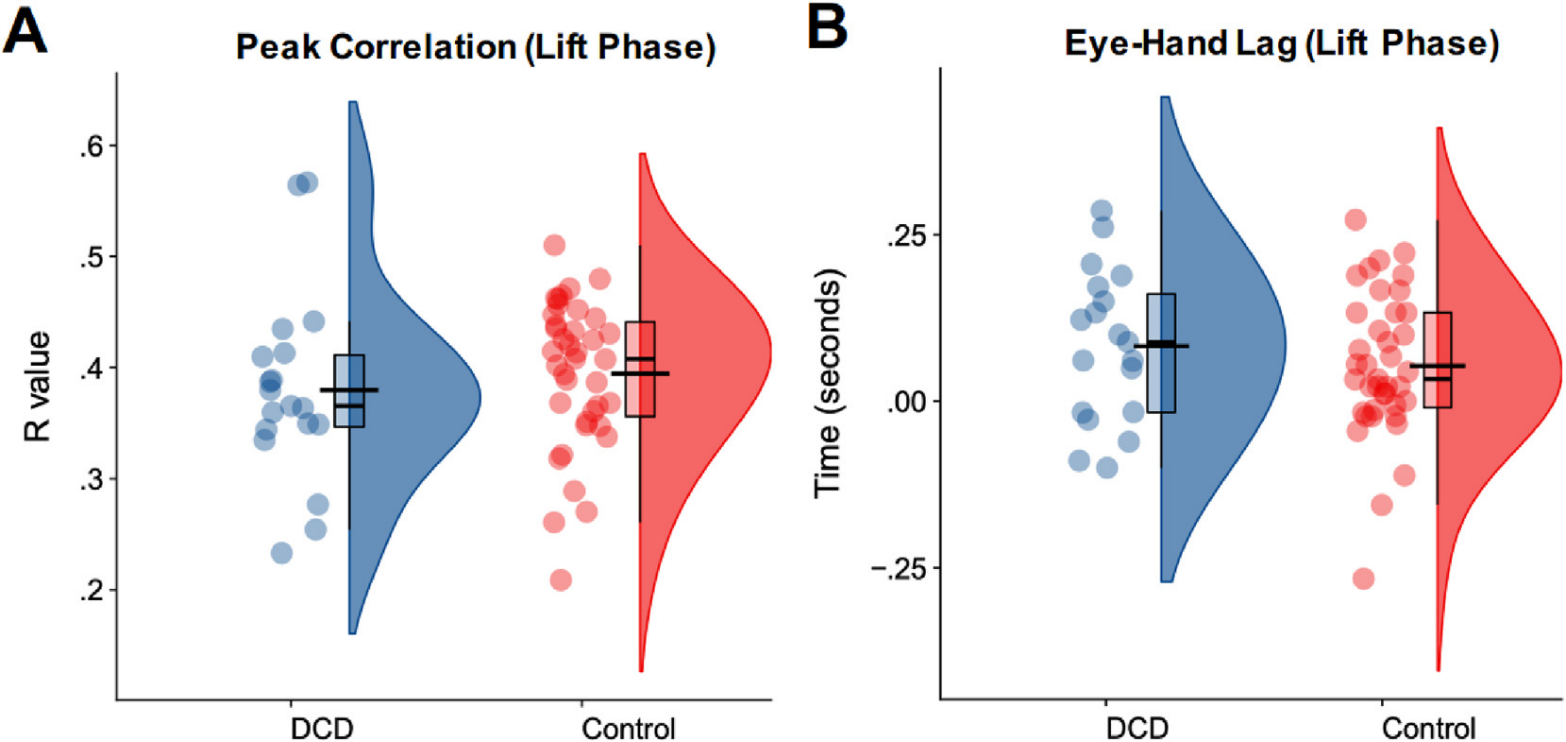
Raincloud plots with individual datapoints, boxplot and half violin plots illustrating the metrics of hand eye coordination for the DCD group (n = 19) compared to the Control group (n = 39). The shorter black bar indicates the median value and the wider black bar indicates the mean.

**Fig. 3:**
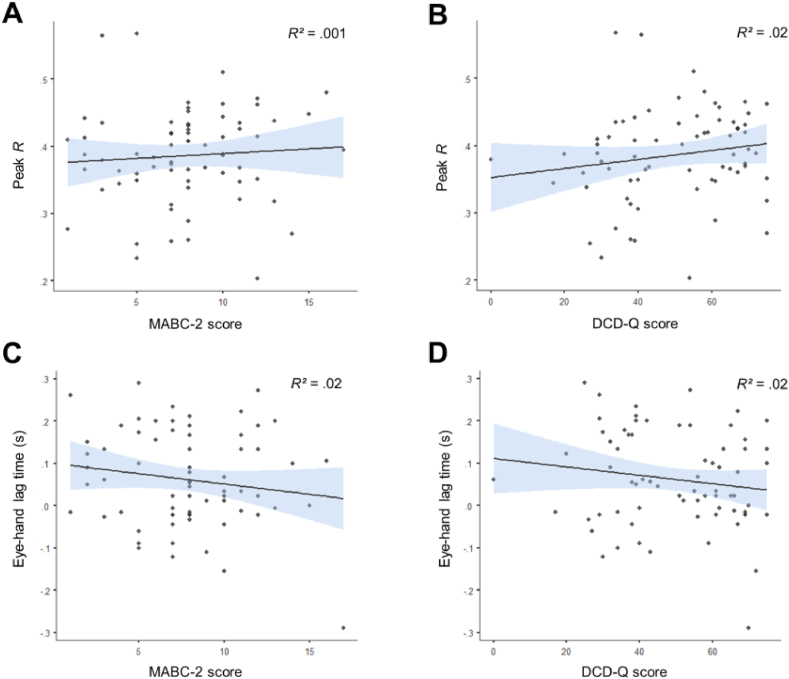
Scatterplots showing the relationship between Peak R with the MABC-2 scores (A) and the DCD-Q scores (B), as well as hand-eye lag with the MABC-2 scores (C) and the DCD-Q scores (D). Blue shading represents 95% confidence intervals around the line of best fit.

Our main hypothesis was that children with DCD would show a lower degree of sensorimotor prediction than their typically developing counterparts. To test this, we derived two measures of hand-eye coordination – the peak covariation between the hand and eye signals (Peak R) and the temporal disparity between the hand and eye signals (hand-eye lag). Both of these measures were compared between the groups using separate independent samples *t* tests. In terms of Peak R, we found no difference between the DCD and Control groups [.38 *vs* .39; t(56) = .73, *p* = .47, d = .20, BF_10_ = .35; Fig. 2A]. In terms of hand-eye lag, there was also no evidence of a difference between the DCD and Control groups [.08 *vs* .05; t(56) = .96, *p* = .34, d = .27, BF_10_ = .41; Fig. 2B]. Moreover, across the entire sample of 70 individuals (i.e., including participants’ whose MABC-2 scores fell between the 5th and 15th percentile), these variables were not significantly related to either MABC-2 (Peak R: R = .07, *p* = .55, BF_10_ = .18, Fig. 3A; hand-eye lag: R = −.16, *p* = .20, BF_10_ = .33; Fig. 3C) or DCD-Q (Peak R: R = .16, *p* = .18, BF_10_ = .37, Fig. 3B; hand-eye lag: R = −.15, *p* = .21, BF_10_ = .32, Fig. 3D) scores. We thus find no support for our primary hypothesis.

### 2.4 Exploratory analysis

**Fig. 4:**
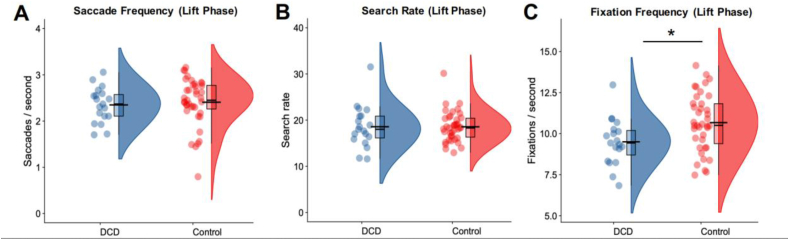
Raincloud plots with individual datapoints, boxplots, and half violin plots illustrating the metrics of gaze behaviour for the DCD group (n = 19) compared to the Control group (n = 39) during the lift phase. The shorter black bar indicates the median value and the wider black bar indicates the mean. ∗ indicates a significant difference at the level of .05.

**Fig. 5:**
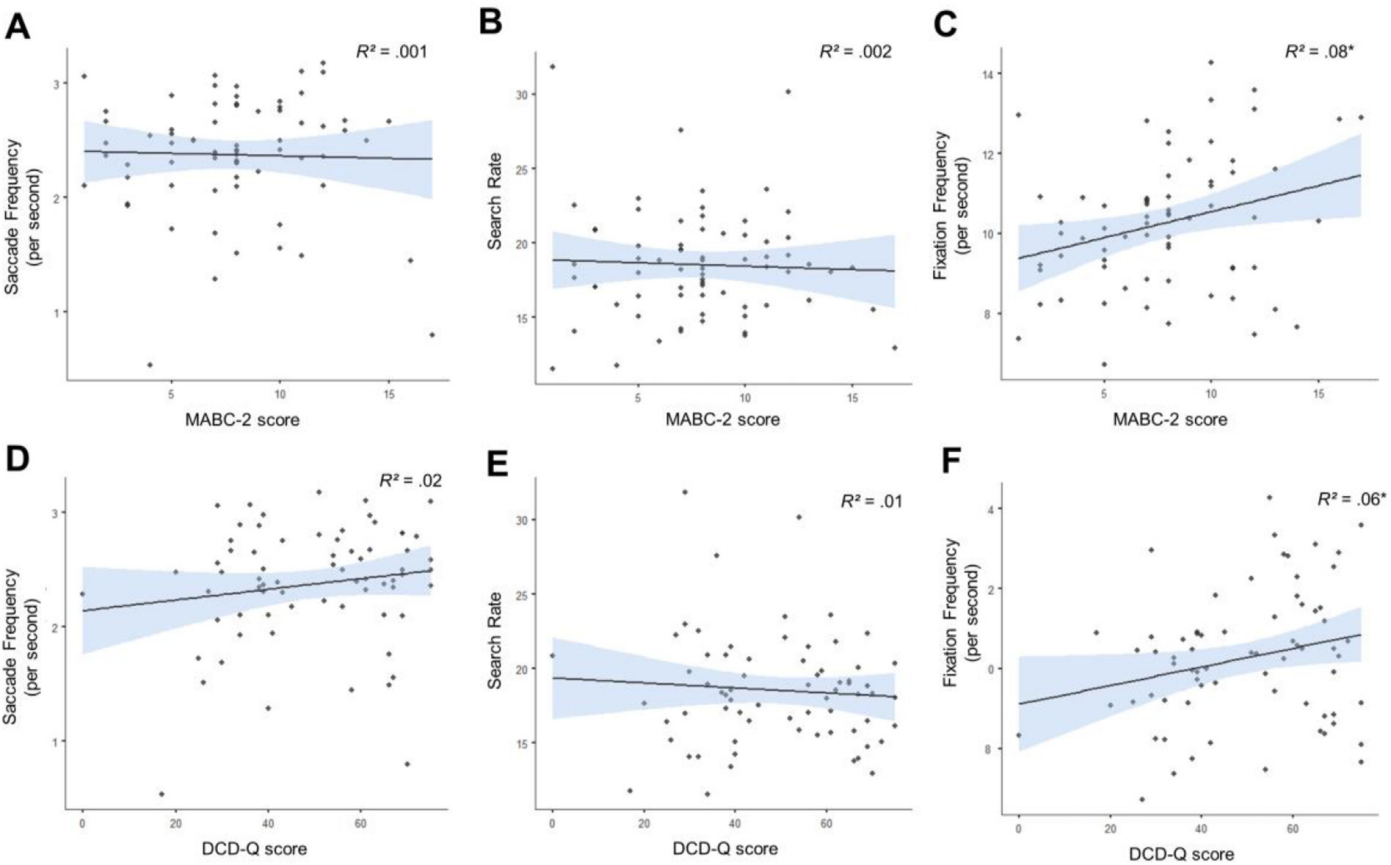
Correlations between the various metrics of gaze behaviour during the lift phase of the task with the MABC-2 scores (A–C) and the DCD-Q scores (D–F). ∗ indicates a significant correlation at the level of .05. Blue shading represents 95% confidence intervals around the line of best fit.

In addition to our main analyses to examine hand-eye coordination between the groups, we also conducted an exploratory analysis to examine how other metrics of gaze behaviour during the lifting phase of the task might vary between the groups. We first compared saccade frequency data between the DCD and control groups. As this dataset showed a negative skew (Shapiro–Wilk test *p* < .001), a Mann–Whitney *U* test found no difference between the DCD and control groups (2.29 *vs* 2.41; W = 432.00, *p* = .32, Rank–Biserial Correlation = .166; Fig. 4A). Furthermore, there were no significant associations between saccade frequency and either MABC-2 (r_s_ = .10, *p* = .39, BF_10_ = .15; Fig. 5A) or DCD-Q (r_s_ = .14, *p* = .26, BF_10_ = .32; Fig. 5D) scores respectively.

Next, we examined fixation search rate, which also deviated from normality (Shapiro–Wilk test *p* = .004). As with saccade frequency, we also found no difference between the groups 18.57 *vs* 18.57; W = 378, *p* = .91, Rank–Biserial correlation = .02, BF_10_ = .45; Fig. 4B, and no significant associations (MABC-2: r_s_ = .02, *p* = .90, BF_10_ = .16, Fig. 5B; DCD-Q: r_s_ = −.08, *p* = .50, BF_10_ = .18, Fig. 5E).

Finally, we examined fixation frequency during the lifting phase of the task with an independent-samples *t*-test. Here, we observed that participants in the DCD group tended to have fewer fixations per second than participants in the control group [9.50 *vs* 10.67; t(56) = 2.54, *p* = .01, d = .71, BF_10_ = 3.74; Fig. 4C]. These effects were consistent with the full–sample correlation analyses, where fixation frequency showed weak, positive associations with both MABC-2 (R = .28, *p* = .02, BF_10_ = 2.38, Fig. 5C) and DCD-Q (R = .24, *p* = .04, BF_10_ = 1.01, Fig. 5F).

## 3 Discussion

Significant debate surrounds the underlying cause of DCD. One hypothesis posits that DCD reflects a specific impairment in using, or difference in the nature of the utilization of, prediction for sensorimotor control (P. H. Wilson et al., 2013). The current study aimed to examine whether children with DCD coordinate the eyes and hand in a less-predictive fashion than typically developing counterparts, and thus determine whether these metrics of hand-eye coordination might serve as an index of this disorder.

In our sample, we found no evidence to support the proposition that children with DCD coordinate their hands and eyes differently than children without DCD. Our measure of the relationship between the eye and the hand over time when lifting an object (peak covariation between vertical hand and eye signals, or Peak R) showed no hint of a difference between our groups. Similarly, both groups had a very similar length of lag between the hand and the eye. It is worth noting that, with the latter measure, our control group's eye led their hand by such a short duration (~50 msec) that there was little opportunity for a smaller lag to be observed. Furthermore, our sample size would only have permitted the detection of a relatively large effect size, and thus more subtle differences between the groups in these metrics might still exist. Given the nature and magnitude of the differences between children with and without DCD in a range of other skilled manual behaviours (e.g., as indexed by the robust differences between the groups in terms of both the MABC-2 scores measured in the lab, and the real-world retrospective observations provided by participants' parents in the DCD-Q; see Table 1), we would have presumed that any effects relevant to the aetiology of DCD might be reasonably large. The other main point of caution with this dataset is the presence of co-occurring developmental conditions in our DCD group. Although we had no diagnostic verification, our questionnaires to assess ASD and ADHD traits suggested that the DCD group had a substantially higher level of both of these conditions than the Control group (Table 1). Using the criteria recommended by these measures, 9 out of our 19 participants with DCD also met the criteria for Autism (scoring over 76 on the Autism Spectrum Quotient: Children's Version), with a further two also appearing to have ADHD (scoring over 97 on the ADHD rating Scale-IV). It is possible that these co-occurring conditions might have masked any latent differences between the groups, but given that the considerable overlaps between these phenotypes is well-established (Sumner et al., 2016), we shall not attempt to separate these out in our modest sample (please note that all data, including these metrics, are available online: https://osf.io/fm247/).

Our findings are not supportive of theories proposing a generic deficit in sensorimotor prediction as the underpinning factor of DCD (P.H. Wilson et al., 2013a, Wilson et al., 2013b). And, while our findings might indeed seem limited in comparison to the reasonably large body of work which supports this view (Adams et al., 2014), it is undertaken in a context which is not ostensibly challenging and frustrating for participants – a factor which is widely acknowledged in the extant literature on sensorimotor control in DCD as a potential confounding factor (Bhoyroo et al., 2018; Parr et al., 2020). Indeed, lifting and interacting with objects for the purpose of experiencing their properties is one of the few motor tasks which is not explicitly taught and/or learned, and is likely undertaken just as much in children with DCD as those without DCD, in stark contrast to the tasks traditionally used to examine prediction in this group (e.g., double-step reaching, ball catching, etc.). Notably, although differences in hand-eye coordination are usually observed in DCD (Wilmut et al., 2006, 2007; Wilmut & Wann, 2008; Wilson et al., 2013a, Wilson et al., 2013b; Warlop et al., 2020), similar null effects have been observed during simple object interaction and grasping task variants (Sellami, 2008; Wilmut & Wann, 2008). Consequently, it is possible that sensorimotor coordination deficits in DCD implicate context-sensitive mechanisms that are affected by levels of task complexity and/or experience. These findings do not offer any alternative insights into the underlying causes of DCD, however they could motivate future work examining how predictive information is integrated with sensory input under different environmental conditions, as has recently been done in Autism Spectrum Disorder (Arthur et al., 2020; Palmer et al., 2013, 2015).

This study is the first to our knowledge to examine hand-eye coordination while lifting an object in young children. Although there is very little work studying this, it is worth acknowledging that the lag between the hand and the eye was small, with many individuals across both groups showing no discernible lag and even an inverse lag with the eye following the hand (Fig. 2B). These data might suggest that many individuals in this age group tend to lift objects in a reasonably feedback-driven fashion, using visual information to undertake the movement. Indeed, these closely-matched signatures are consistent with research showing early-flight visual cues can aid the perception of object weight (e.g., Hamilton et al., 2007). Therefore, this suggests that the ‘top-down’, goal-directed integration of sensorimotor systems appears optimal in both groups during this task. Future work might examine whether these apparent hand-eye coordination patterns emerge in children with DCD during more target-directed object placement or obstacle avoidance tasks (Johansson et al., 2001; Lavoie et al., 2018).

The final point to discuss is the exploratory analyses yielding the finding that child with DCD have atypical visual sampling behaviours (Fig. 4, Fig. 5, Supplementary Material). Here, tendencies for DCD participants to make a high number of pre-lift fixations led to a later ‘anchoring’ of gaze during their reach-to-grasp movements compared to the control group (Supplementary Figure 1D). Visual sampling differences during these initial, preparatory task stages generally affect subsequent action sequences (Land, 2009), and we indeed observed that participants in our DCD group showed a lower fixation frequency during the later lifting phases of the trial (Fig. 4C). These differences could represent a broad attentional discrepancy related to atypical sampling of the object being interacted with and/or an increased focus on smooth pursuit rather than scan-ahead saccades. Alternatively, they could implicate more complex, dynamic computational mechanisms, which modulate predictive sensorimotor behaviours according to stochastic, goal-relevant environmental variables (e.g., uncertainty, task rewards, energetic costs; see Franklin & Wolpert, 2011 for review). However, as most *p* values for this finding, and the associated correlations, were only marginally significant by conventional thresholds, and BFs were generally indicative of weak or inconclusive evidence for the alternative hypothesis, we suggest that this finding should be replicated in follow-up work to verify its veracity.

## Open practices

The study in this article earned Open Data badge for transparent practices. Materials and data for the study are available at https://osf.io/fm247/.

## Footnotes

2 Our analysis focussed on the lifting portion of the entire reach-lift-hold task as our prior work using this task has indicated that the hand and eye signals are difficult to reconcile (i.e., do not move in concert) during the reaching and the holding phases of the task; See Arthur et al., 2019Supplementary Figure 1.
