# Supplementary material for: Visuo-motor attention during object interaction in children with developmental coordination disorder
Source: Cortex. 2021 May;138:318–28. doi: 10.1016/j.cortex.2021.02.013 (PMC8064026; doi:10.1016/j.cortex.2021.02.013)
Supplement: Multimedia component 1 [file mmc1.docx]

**Supplementary Materials**

Reach phase: Gaze data

Following the findings that children with DCD utilise atypical visual sampling strategies in the task (Figures 4-5), we examined whether gaze behaviours differed between groups during the *reach-to-grasp* phase. Here, saccade frequency, fixation search rate, and fixation frequency were extracted, as in the main text. All of these variables deviated from normality (Shapiro-Wilk test: *p*’s<.001), and non-parametric tests were employed. Though Mann-Whitney U comparisons indicated that saccade frequency did not differ between groups (W=301, p=.25, Rank-Biserial correlation=.19, BF_10_=0.49; Supplementary Figure 1A), DCD participants showed significantly higher gaze search rates (W=243, p=.035, Rank-Biserial correlation=.34, BF_10_=0.82; Supplementary Figure 1B) and fixation frequencies (W=246, p=.04, Rank-Biserial correlation=.34, BF_10_=1.10; Supplementary Figure 1C). Furthermore, there were significant associations between DCD-Q scores and saccade frequency (r_s_= -.31, p=.01, BF_10_= 3.96; Supplementary Figure 2B), search rate (r_s_= -.25, p=.035, BF_10_= 1.30; Supplementary Figure 2D), and fixation frequency (r_s_= -.31, p=.009, BF_10_= 4.17; Supplementary Figure 2F). Similar MABC-2 correlations emerged for saccade (r_s_ = -0.26, p=.03, BF_10_= 0.46; Supplementary Figure 2A) and fixation frequency (r_s_ = -0.35, p=.003, BF_10_= 6.44; Supplementary Figure 2E), however associations did not reach statistical significance between MABC-2 scores and gaze search rate (r_s_ = -0.22, p=.07, BF_10_= 1.59; Supplementary Figure 2C). Together, these exploratory results suggest that the DCD group may be using atypical visual sampling strategies prior to making contact with the object.

Next, we explored whether these potential differences in visual sampling affected the ‘anchoring’ of gaze during participants’ reaching actions. Here, we assessed the onset of the final pre-object contact fixation, as gaze is typically fixed on goal-directed targets during reach-to-grasp movements (Neggers & Bekkering, 2001). Mann-Whitney U test indicated that the onset of this fixation (Shapiro-Wilk test p<.001) was significantly later in the DCD group (M=-1.05) compared to the control group (M=-1.34) (W=243.5, p=.036, Rank-Biserial correlation=.34, BF_10_=1.49; Supplementary Figure 1D). This suggests that atypical visual sampling strategies may have disrupted goal-directed ‘anchoring’ of gaze in participants with DCD. However, correlations were inconsistent for this measure, with a marginally significant association emerging with MABC-2 (r_s_ = -0.24, p=.05, BF_10_= 0.61; Supplementary Figure 2G) but not DCD-Q (r_s_= -.23, p=.06, BF_10_= 0.83; Supplementary Figure 2H) scores.


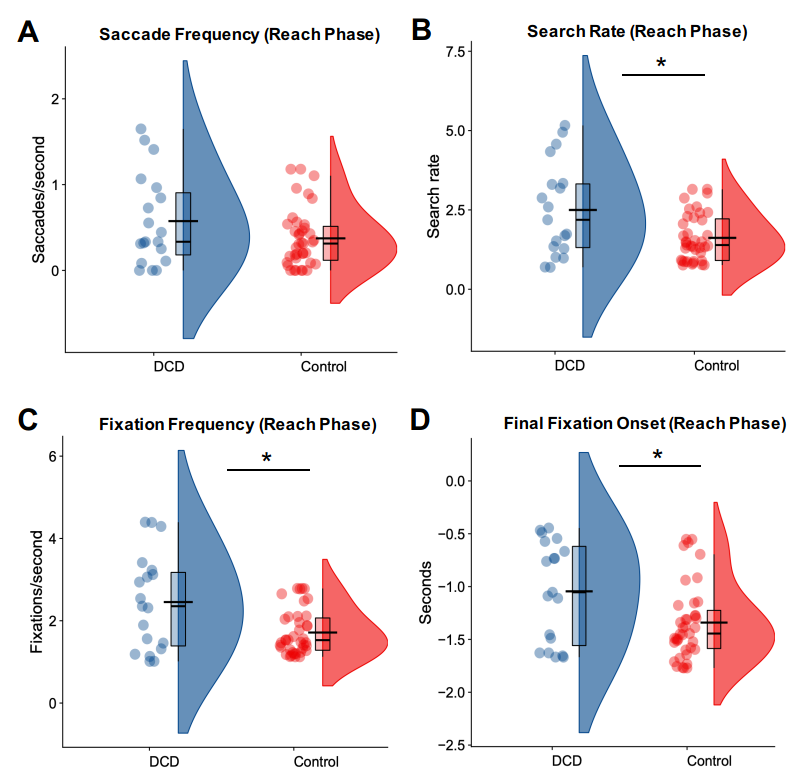


**Supplementary Figure 1.** Raincloud plots with individual datapoints, boxplot and half violin plots illustrating the metrics of gaze behaviour for the DCD group (n=19) compared to the Control group (n=39) during the reach phase. The shorter black bar indicates the median value and the wider black bar indicates the mean. * indicates a significant difference at the level of .05.


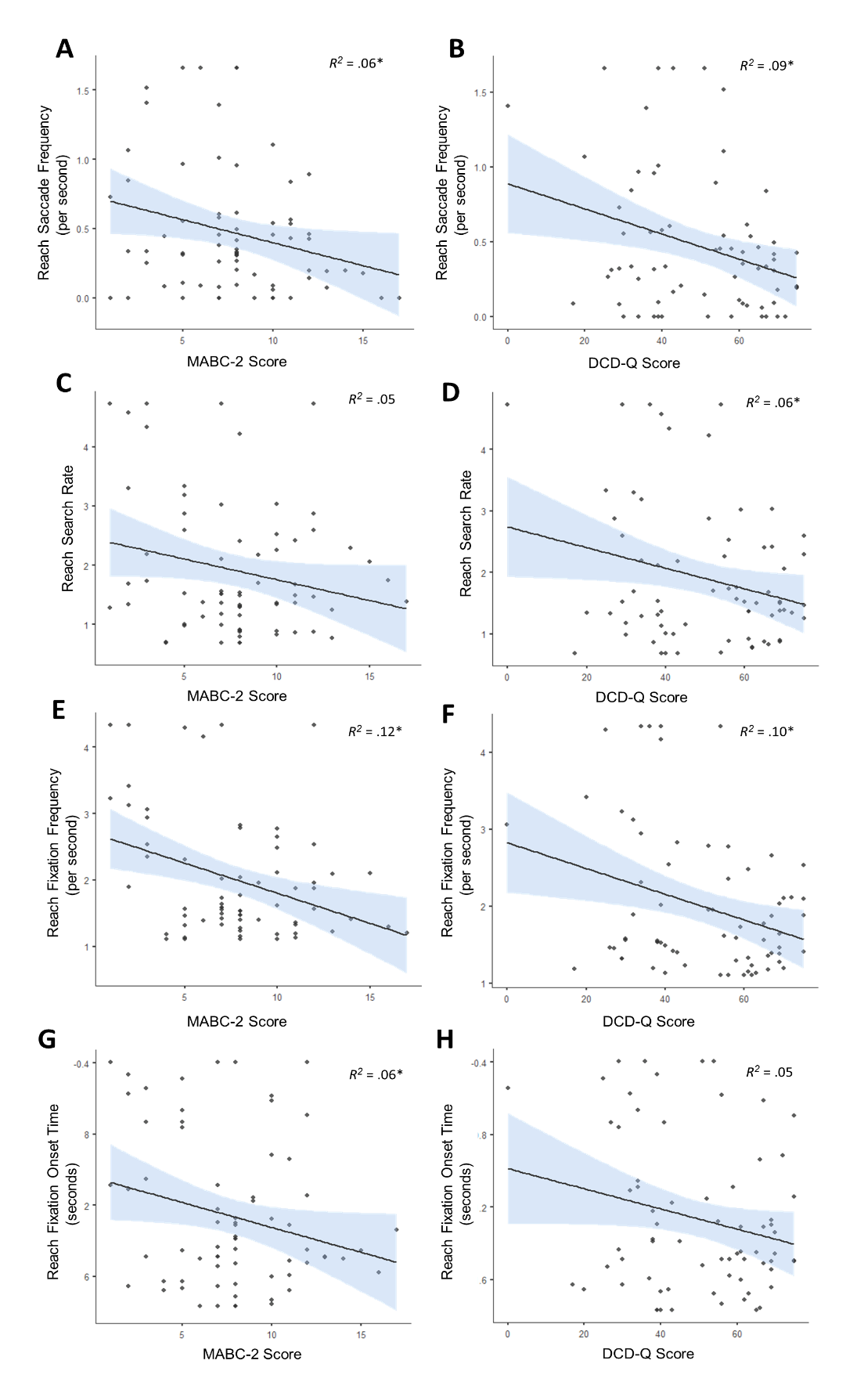


**Supplementary Figure 2.** Correlations between the various metrics of gaze behaviour during the reach phase of the task with the MABC-2 scores (A, C, E, G) and the DCD-Q scores (B, D, F, H). * indicates a significant correlation at the level of .05. Blue shading represents 95% confidence intervals around the line of best fit.

Reach phase: Kinematic data

Finally, given the atypical visual sampling behaviours noted above, we examined whether any differences in reach kinematics subsequently emerged between groups. Though movement durations were normally distributed, peak hand velocity, and the time taken to reach this landmark, were positively skewed (Shapiro-Wilk test p<.001). T-tests showed no differences in movement duration between the DCD and control groups (t(56)=1.44, p=.16, d=0.40, BF_10_=0.65; Supplementary Figure 3A) and there were no significant associations for this measure (MABC-2: r_s_ = .03, p=.81, BF_10_= 0.15; DCD-Q: r_s_= -.01, p=.95, BF_10_= 0.15). Furthermore, Mann-Whitney U tests indicated that there was no between-group differences in peak hand velocity (W=257, p=.06, Rank-Biserial correlation=.31, BF_10_=1.38; Supplementary Figure 3B) or time to peak hand velocity (W=257, p=.06, Rank-Biserial correlation=.31, BF_10_=0.55; Supplementary Figure 3C). There was a weak, negative association with DCD-Q scores (r_s_= -.27, p=.03, BF_10_= 1.72), however these relationships were not significant for MABC-2 scores (r_s_ = -0.17, p=.16, BF_10_= 1.10). Moreover, there were no significant associations observed for time to peak hand velocity (MABC-2: r_s_ = -.20, p=.09, BF_10_= 0.23; DCD-Q: r_s_= -.20, p=.09, BF_10_= 0.61). Therefore, no DCD-related differences in hand kinematics were detected during the reach-to-grasp portion of the task.


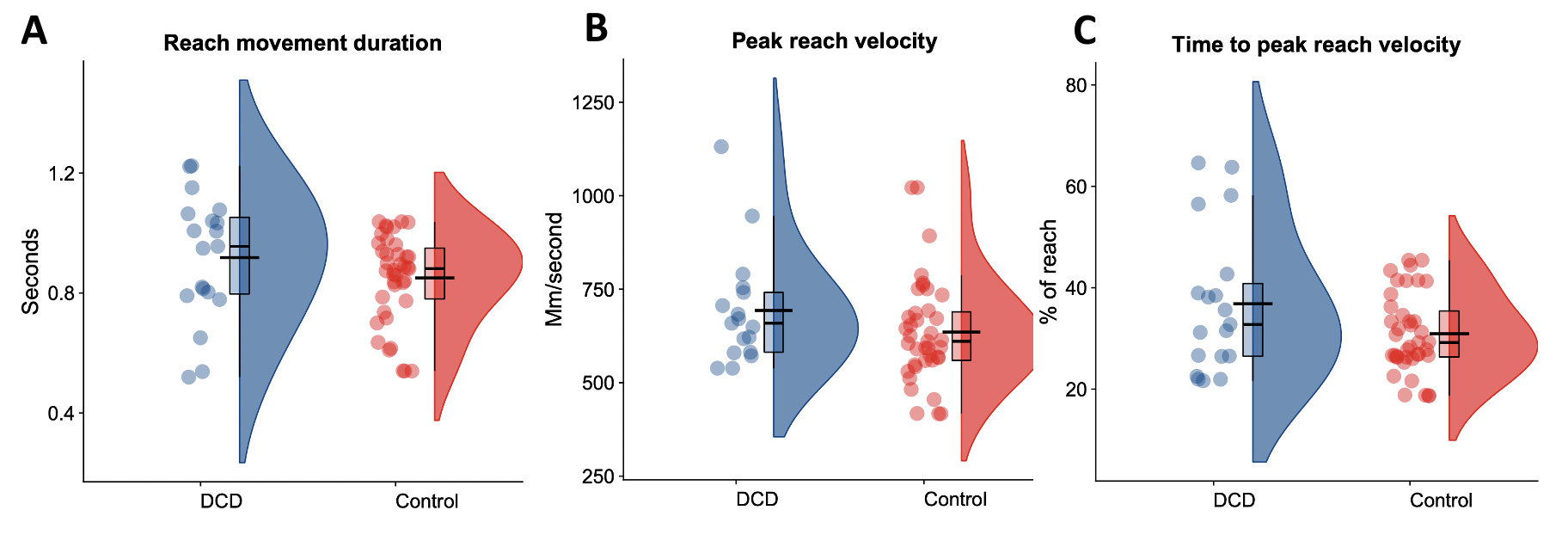


**Supplementary Figure 3.** Raincloud plots with individual datapoints, boxplot and half violin plots illustrating the metrics of gaze behaviour for the DCD group (n=19) compared to the Control group (n=39) during the reach phase. The shorter black bar indicates the median value and the wider black bar indicates the mean.
